# Alisol A Exerts Anti-Proliferative Activity against Human Oral Cancer Cells through Triggering JNK/p38 MAPK-Mediated Apoptotic Cascade

**DOI:** 10.32604/or.2025.069877

**Published:** 2025-10-22

**Authors:** Yi-Tzu Chen, Shao-Hsuan Kao, Chun-Yi Chuang, Chun-Wen Su, Wei-En Yang, Chih-Hsin Tang, Shun-Fa Yang, Chiao-Wen Lin

**Affiliations:** 1 School of Dentistry, Chung Shan Medical University, Taichung, 402, Taiwan; 2 Institute of Oral Sciences, Chung Shan Medical University, Taichung, 402, Taiwan; 3 Department of Dentistry, Chung Shan Medical University Hospital, Taichung, 402, Taiwan; 4 Institute of Medicine, Chung Shan Medical University, Taichung, 402, Taiwan; 5 Department of Medical Research, Chung Shan Medical University Hospital, Taichung, 402, Taiwan; 6 School of Medicine, Chung Shan Medical University, Taichung, 402, Taiwan; 7 Department of Otolaryngology, Chung Shan Medical University Hospital, Taichung, 402, Taiwan; 8 Department of Pharmacology, School of Medicine, China Medical University, Taichung, 404, Taiwan; 9 Department of Medical Laboratory Science and Biotechnology, Asia University, Taichung, 413, Taiwan; 10 Chinese Medicine Research Center, China Medical University, Taichung, 404, Taiwan

**Keywords:** Alisol A, oral squamous cancer cell, apoptosis, c-Jun N-terminal kinase, p38

## Abstract

**Background:**

Alisol A is a natural compound isolated from *Alismatis Rhizoma*, known for its diverse pharmacological activities, including anticancer and neuroprotective effects. This study aimed to explore the anticancer effects of Alisol A on oral cancer cells and elucidate its underlying mechanisms.

**Methods:**

Cell viability was measured by MTT assay, cell cycle by flow cytometry, and apoptosis by Annexin V/PI staining and caspase activation. Regulation of signaling pathways was analyzed using an apoptosis-related protein array, immunoblotting, and specific kinase inhibitors.

**Results:**

Alisol A reduced the viability of oral cancer cell lines, induced sub-G1 phase accumulation, and augmented the number of apoptotic cells. Protein array results indicated that Alisol A enhanced the expression of heme oxygenase-1 (HO-1), while suppressing cellular inhibitor of apoptosis protein 1 (cIAP1) and X-linked inhibitor of apoptosis protein (XIAP) levels in SCC-9 cells. These changes were further confirmed in both SCC-9 and HSC-3 cells by immunoblotting. In addition, Alisol A triggered the activation of caspase-8, -9, and -3, as well as poly (ADP-ribose) polymerase (PARP) cleavage in both cell lines. Analysis of signaling pathways showed that mitogen-activated protein kinases (MAPKs) were significantly activated by Alisol A. Notably, inhibition of JNK and p38 markedly reduced Alisol A-induced activation of caspase-8, -9, and -3.

**Conclusions:**

Our findings demonstrate that Alisol A exerts potent anticancer effects on oral cancer cells by inducing caspase-dependent apoptosis via activation of the JNK and p38 signaling pathways. These results suggest that Alisol A may have therapeutic potential for the treatment of oral cancer.

## 1 Introduction

Oral cancer is the most common form of head and neck malignancy and is a significant contributor to high mortality rates [1]. Based on the “Global Cancer Statistics 2022” survey, oral cancer has become one of the most common malignant tumors in the world [1]. Among the various subtypes of oral cancer, oral squamous cell carcinoma (OSCC) is responsible for approximately 90% of all oral malignancies. It is most frequently observed in areas such as the tongue, upper and lower gums, the floor of the mouth, the upper jaw, and the buccal mucosa [2]. Furthermore, OSCC exhibits a high level of invasiveness, and its metastasis starts early [3]. The primary risk factors associated with oral cancer encompass a range of behaviors and conditions, including the frequent consumption of betel nut, long-term smoking, heavy alcohol consumption, chronic viral infections, exposure to radiation, and unhealthy dietary patterns [4]. Currently, the primary approaches to treating oral cancer involve surgical procedures coupled with radiation therapy, chemotherapy, or targeted therapy. Nevertheless, the striking recurrence rates and the highly metastatic tendency of oral cancer continue to lead to a poor prognosis and a significant number of patient fatalities [5,6].

Over the past decades, various biological activities of natural components have been widely explored for their health-promoting and potential therapeutic benefits, particularly in cancer treatments [7,8]. *Alisma orientale* (Sam.) Juzep. (*Alismataceae*) is a common and popular medicinal herb that has been used in treating nephropathy and promoting urological functions [9]. By modern pharmacological evaluation, a wide spectrum of active constituents from *Alisma orientale* has been identified and characterized with diverse biological activities, including anti-atherosclerotic, antinephritic, and immunomodulatory effects [10]. Among the active constituents from rhizomes of *Alisma orientale*, Alisol A is a proalkane-type tetracyclic triterpenoid with potential anticancer activity against colorectal cancer cells, breast cancer cells, and nasopharyngeal carcinoma cells [11–13]. However, whether Alisol A exhibits anticancer effects on oral cancer cells and the underlying mechanistic act is not fully explored.

In the present study, we aimed to further investigate the anticancer effect of Alisol A on human oral cancer cells. The influence of Alisol A on cell survival and cellular physiology was explored. Molecular acts triggered by Alisol A were demonstrated by apoptosis-associated protein array analysis and signaling evaluation with specific inhibitors.

## 2 Materials and Methods

### 2.1 Chemicals, Reagents, and Antibodies

General chemicals and reagents, including Alisol A (cat.no. PHL83835), U0126 [extracellular signal-regulated kinase (ERK) inhibitor] (cat.no. 662009), JNK-IN-8 [c-Jun N-terminal kinase (JNK) inhibitor] (cat.no. 420150), and SB203580 [p38 mitogen-activated protein kinase (p38) inhibitor] (cat.no. 559389) were purchased from Sigma-Aldrich (St. Louis, MO, USA). The specific antibodies information: anti-HO-1 (1:10,000, cat.no. ab68477, Abcam, Cambridge, UK), anti-c-IAP1 (1:1000, cat.no. #7065, CST, Danvers, MA, USA), anti-XIAP (1:1000, cat.no. #2045, CST), anti-caspase-8 (1:1000, cat.no. #9746, CST), anti-caspase-9 (1:1000, cat.no. #9502, CST), anti-caspase-3 (1:1000, cat.no. 610323, BD), anti-poly (ADP-ribose) polymerase (PARP) (1:1000, cat.no. #9542, CST), anti-cleaved caspase-8 (1:1000, cat.no. #9496, CST), anti-cleaved caspase-9 (1:1000, cat.no. #9505, CST), anti-cleaved caspase-3 (1:1000, cat.no. #9664, CST), anti-phospho (p)-ERK (1:2000, cat.no. #4370, CST), anti-ERK (1:1000, cat.no. #9102), anti-p-JNK (1:1000, cat.no. #4668), anti-JNK (1:1000, cat.no. #9258, CST), anti-p-p38 (1:1000, cat.no. sc-166182, Santa Cruz, Dallas, TX, USA), anti-p38 (1:1000, cat.no. sc-7972, Santa Cruz), anti-human β-actin (1:10,000, cat.no. ab8226, Abcam), mouse HRP conjugated secondary antibodies (1:1000, cat.no. #7076, CST), and rabbit HRP conjugated secondary antibodies (1:1000, cat.no. #7074, CST).

### 2.2 Cells and Cell Culture

SCC-9 (cat.no. CRL-1629), and HSC-3 (cat.no. JCRB0623) cells were acquired from the American Type Culture Collection (Manassas, VA, USA) and the Japanese Collection of Research Bioresources (Osaka, Japan), respectively. For maintenance, SCC-9 cells were cultured in 1:1 DMEM/F12 medium, supplemented 10% fetal bovine serum (FBS), 2.5 mM L-glutamine, 1% penicillin/streptomycin, 0.5 mM sodium pyruvate, 15 mM HEPES, 400 ng/mL hydrocortisone, and 0.1 mM nonessential amino acids (Life Technologies, Carlsbad, CA, USA). HSC-3 cells were maintained in high-glucose DMEM (Gibco BRL, New York, NY, USA) containing 10% fetal bovine serum and 1% penicillin-streptomycin. Cultures were kept at 37°C in a 5% CO_2_ humidified atmosphere. All cell lines are short tandem repeat (STR) authenticated. Mycoplasma contamination was routinely tested by PCR analysis.

### 2.3 Cell Viability Analysis

SCC-9 and HSC-3 cells were grown to 80% confluency, and then seeded into 24-well plates at 2 × 10^4^ cells/mL. After 16 h (h) incubation, the cells were incubated with Alisol A (dissolved in DMSO) at serial concentrations (0, 25, 50, 75 and 100 μM) for 24 h. The treated cells were washed with 1X PBS (pH 7.4) and reacted with 5 mg/mL MTT solution (#L11939-06; Alfa Aesar, Ward Hill, MA, USA) at 37°C for 4 h. After being washed with PBS, the cells were incubated with isopropanol to dissolve formazan. The relative level of formazan was determined by measuring absorbance at 563 nm using a microplate reader (MQX200; Bio-Tek Instruments, Winooski, VT, USA) [14].

### 2.4 Evaluation of Cell Cycle Distribution

After treatments with different concentrations of Alisol A (0, 25, 50, 75 and 100 μM) for 24 h, SCC-9 and HSC-3 cells were washed with 1X PBS (pH 7.4), fixed in 70% ethanol at −20°C for 16 h, and then reacted with PI staining buffer (#550825; BD Biosciences, Milpitas, CA, USA) with RNase at 25°C for 15 min in the dark. The stained cells were analyzed by a flow cytometer (Accuri C6 Plus flow cytometer, BD Biosciences, San Diego, CA, USA) to assess the cell cycle distribution. Incubation with complete medium was used as a control (indicated as 0 μM). Each cell cycle phase was presented as a percentage of gated cells.

### 2.5 PI/Annexin V Staining for Apoptosis

To evaluate cell apoptosis in response to Alisol A, SCC-9 and HSC-3 cells treated with different concentrations of Alisol A (0, 25, 50, 75 and 100 μM) for 24 h were incubated with PI/Annexin V-FITC(#556547; BD Biosciences, Milpitas, CA, USA) for 15 min in the dark, and then analyzed by a flow cytometer (Accuri C6 Plus flow cytometer, BD Biosciences, San Diego, CA, USA). Non-treated cells incubated with PI/Annexin V-FITC were used as negative controls. Cells exhibiting PI-high/Annexin V-low and PI-high/Annexin V-high represented the early and late stages of apoptosis, respectively.

### 2.6 Expression Profiles of Apoptosis-Associated Proteins

T to assess apoptosis-related protein expression in oral cancer cells, SCC-9 and HSC-3 cells were exposed to 100 μM Alisol A for 24 h After treatment, the cells were washed twice with cold 1X PBS (pH 7.4) and lysed using the lysis buffer provided in the Proteome Profiler™ Human Apoptosis Array Kit (cat.no. ARY009. R&D Systems, Minneapolis, MN, USA). This protein array is capable of detecting 35 different human apoptosis-related proteins. The signal from each spot on the array was visualized using a Luminescent Image Analyzer (LAS 4000 mini, GE Healthcare Bio-Sciences, Fuji, Tokyo, Japan), and the signal intensity was quantified using Image-Pro Plus software V6 (Fuji, Tokyo, Japan). Each protein spot intensity was normalized to the reference spots on the array and then to the vehicle-treated control group to determine relative expression changes.

### 2.7 Immunoblotting

To further confirm protein expression levels, Western blot analysis was performed. After treatments with Alisol A, or preincubated with 5 μM U0126 (ERK inhibitor), 1 μM JNK-in-8 (JNK inhibitor), or 10 μM SB203580 (p38 inhibitor) for 2 h, reacted with 100 μM Alisol A for 24 h, the treated and control cells were harvested and lysed in ice-cold RIPA buffer (cat.no. 89900, Thermo Fisher Scientific, Waltham, MA, USA). Cell lysates were spun at 14,000× *g* for 15 min at 4°C to eliminate debris, and protein levels in the resulting supernatants were cat.no. (cat.no.#5000006, Bio-Rad Laboratories, Hercules, CA, USA). Protein samples (20–30 μg) were separated using 12.5% SDS-PAGE and then transferred to PVDF membranes (Millipore, Billerica, MA, USA). The membranes were incubated in 5% (w/v) BSA prepared in PBS for 1 h at room temperature to block non-specific binding, followed by incubation with specific primary antibodies against target apoptosis-related proteins at 4°C overnight. After washing with PBST (PBS containing 0.5% Tween-20), membranes were incubated with HRP-conjugated secondary antibodies for 1 h at room temperature. Detection was carried out using enhanced chemiluminescence (ECL) reagent (cat.no. WBKLS0500, Millipore, Burlington, MA, USA), and signals were captured using the LAS 4000 mini luminescence imaging system (Fuji, Tokyo, Japan).

### 2.8 Statistical Analysis

Data from three independent experiments were used for statistical analysis. The analysis results were expressed as the mean ± standard deviation (SD). The Student’s *t*-test was conducted to evaluate whether the changes between the two groups were significant by using Sigma Plot (v.14.0) (San Jose, CA, USA). *p* < 0.05 was considered statistically significant.

## 3 Results

### 3.1 Alisol A Dampens the Cell Viability of Human Oral Cancer Cells

**Figure 1:**
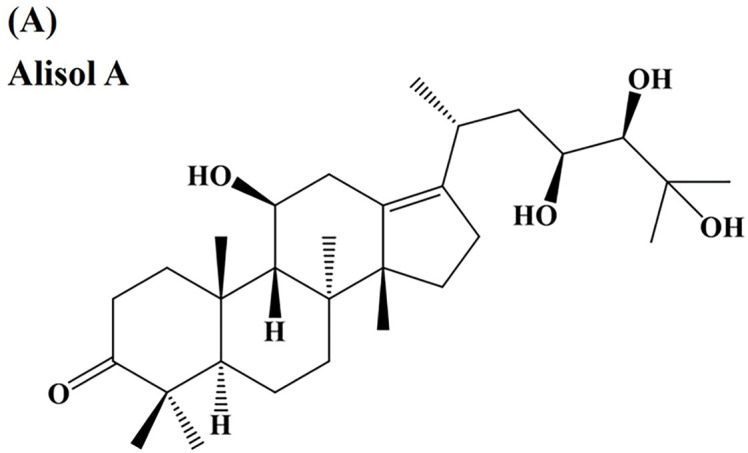

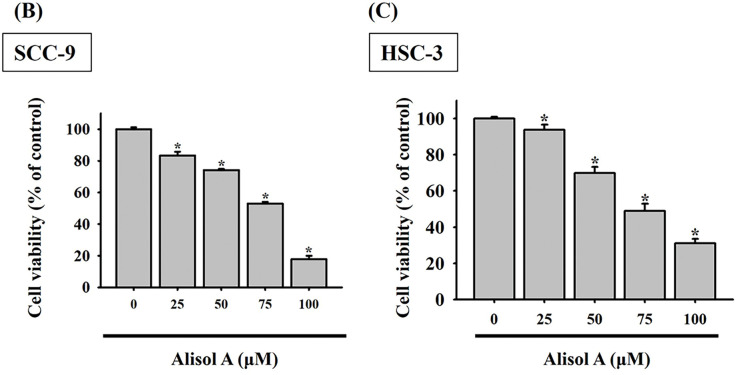
Inhibitory effect of Alisol A on cell viability of oral cancer cells. (**A**) The chemical structure of Alisol A. (**B**) SCC-9 cells and (**C**) HSC-3 cells were incubated with a serial concentration of Alisol A (25, 50, 75 and 100 μM) for 24 h, then subjected to MTT assay to evaluate the cell viability changes. Cell viability was indicated as a percentage of Alisol A treatment/DMSO (0 μM). *p < 0.05 vs. control

Fig. 1A shows the chemical structure of Alisol A. MTT assays were performed to evaluate the impact of Alisol A on the viability of SCC-9 and HSC-3 cells. The findings revealed a significant, dose-dependent reduction in cell viability following Alisol A treatment (*p* < 0.05 compared to control. Alisol A treatment at 100 μM significantly reduced the cell viability of SCC-9 and HSC-3 to 17.8 ± 2.2% and 31.1 ± 2.4% of control, respectively (*p* < 0.05 vs. control) (Fig. 1B,C). Taken together, the results exhibit that Alisol A is able to decrease the viability of human oral cancer cells.

### 3.2 Alisol A Increases Sub-G1 Phase and Reduces G0/G1 Phase in Human Oral Cells

**Figure 2:**
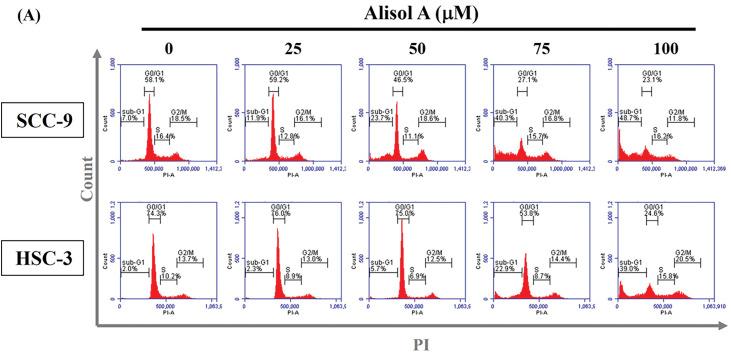

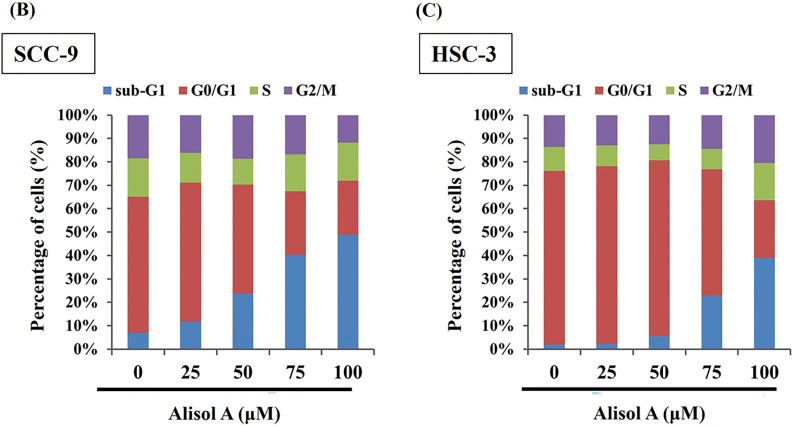
Alisol A altered cell cycle distributions in human oral cancer cells. (**A**) SCC-9 and HSC-3 cells were incubated with serum-free medium for 16 h, reacted with Alisol A at 25, 50, 75 and 100 μM for 24 h, and then subjected to evaluate cell cycle distribution using PI-staining and flow cytometric analysis. (**B,C**) The individual cell cycle phase, including sub-G1, G0/G1, sub-G1, S, and G2/M, was exhibited as a percentage of the total cell count

Subsequently, the impact of Alisol A on cell cycle distribution was assessed through flow cytometry analysis. Our observations revealed that the G0/G1 phase ratio was clearly decreased from 58.1% to 23.1% (SCC-9) and from 74.3% to 24.6% (HSC-3) in response to 100 μM Alisol A treatment (*p* < 0.05 vs. control, Fig. 2A). In parallel to the decrease of G0/G1 phase ratio, Alisol A treatments elevated sub-G1 phase ratio up to 48.7% (SCC-9) and 39.0% (HSC-3), respectively (*p* < 0.05 vs. control, Fig. 2A). Interestingly, G2/M phase ratio was reduced in SCC-9 cells but elevated in HSC-3 cells in response to Alisol A treatments compared to the control. Collectively, these observations show that Alisol A significantly increases the sub-G1 phase and reduces the G0/G1 phase ratio, but differentially influences G2/M phase ratio in SCC-9 and HSC-3 cells (Fig. 2B,C).

### 3.3 Alisol A Induces Apoptosis of Human Oral Cancer Cells

**Figure 3:**
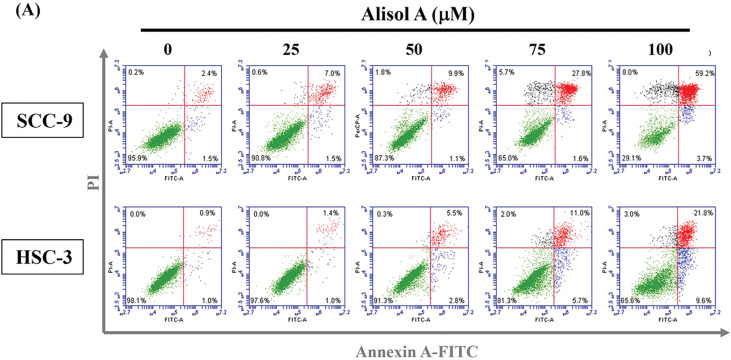

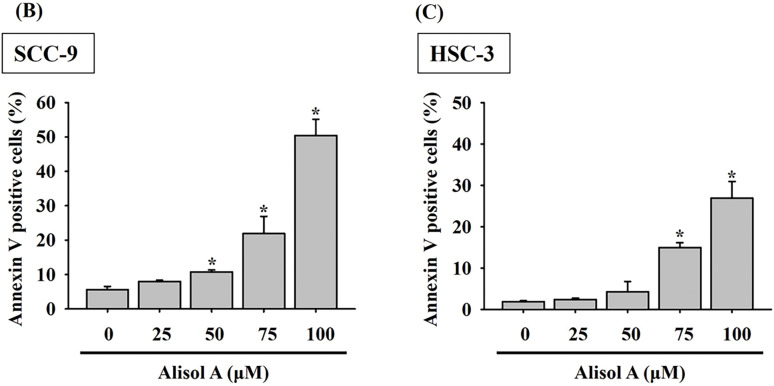
Alisol A triggered apoptosis in human oral cancer cells. (**A**) Cells were reacted with Alisol A at 25, 50, 75 and 100 μM for 24 h, and then subjected to determination of cell cycle distribution using PI/Annexin V-double staining and flow cytometric analysis. (**A**) The representative flow cytometric analyses were presented. (**B,C**) Quantitation of (**B**) SCC-9 and (**C**) HSC-3 cells with high intensity of PI and Annexin V in response to Alisol A. The quantitative data from three independent experiments were exhibited as mean ± SD. *p < 0.05 vs. control

Based on the remarkable increase in the sub-G1 phase, the changes in the number of apoptotic cells in response to Alisol A were next evaluated. As shown in Fig. 3A, most cells (>95%) were PI-low and Annexin V-low in the control group. In contrast to control, Alisol A treatments increased Annexin V-positive cells up to 50.4 ± 4.69% (SCC-9) (Fig. 3B) and 26.9 ± 4.04% (HSC-3) (Fig. 3C), representing the significant induction of apoptosis in oral cancer cells in response to Alisol A treatment (*p* < 0.05 vs. control). These observations reveal that Alisol A significantly provokes cellular apoptosis in oral cancer cells.

### 3.4 Alisol A Upregulates HO-1 and Downregulates cIAP-1 and XIAP in Human Oral Cancer Cells

**Figure 4:**
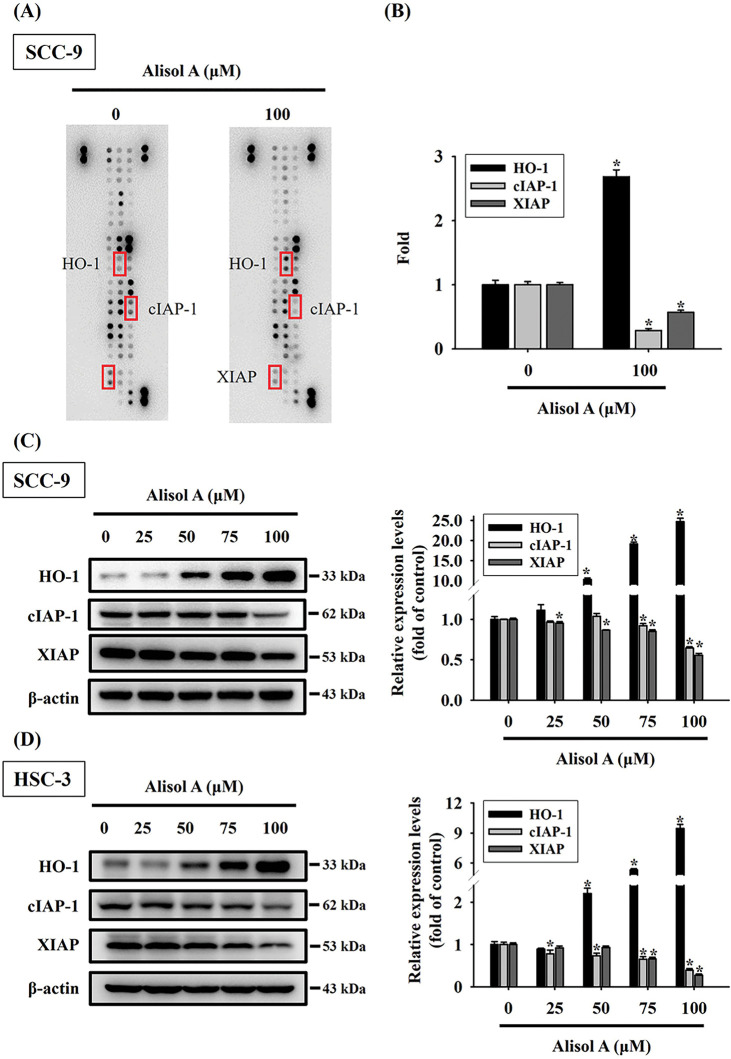
Alisol A induced differential apoptotic protein profiling in human oral cancer cells. (**A**) Cells reacted with 100 μM Alisol A for 24 h were subjected to crude protein extraction and the subsequent apoptotic protein profiling analysis using protein microarrays. Representative changes of apoptotic proteins between control and Alisol A treatment were indicated. (**B**) densitometric analysis for the indicated proteins with changes between control and Alisol A treatment. (**C,D**) Protein expression of HO-1, cIAP1, and XIAP in (**C**) SCC-9 and (**D**) HSC-3 cells was evaluated by immunoblotting. Chemiluminescence intensity was relatively quantitated by densitometric analysis and normalized with β-actin (internal control). The quantitative data from three independent experiments were expressed as mean ± SD. *p < 0.05 vs. control

To further explore the apoptotic mechanism provoked by Alisol A in oral cancer cells, the treated cells were subjected to protein expression profiling using a human apoptotic protein microarray. The resulting protein profiling showed that heme oxygenase-1 (HO-1) was significantly upregulated in SCC-9 cells after Alisol A treatment (100 μM for 24 h), while cIAP-1 and XIAP were down-regulated (Fig. 4A,B). In parallel, the changes of cellular HO-1, cIAP-1, and XIAP levels were also evaluated by immunoblotting, and consistently, the observations revealed that Alisol A contributed to the increase in HO-1 levels and the decrease in cIAP-1 and XIAP levels in SCC-9 and HSC-3 cells (*p* < 0.05 vs. control, Fig. 4C,D). These observations show that Alisol A alters the protein expression of apoptotic signaling components and may consequently trigger the apoptotic cascade in oral cancer cells.

### 3.5 Alisol A Induces Caspase-Mediated Apoptotic Cascade in Oral Cancer Cells

**Figure 5:**
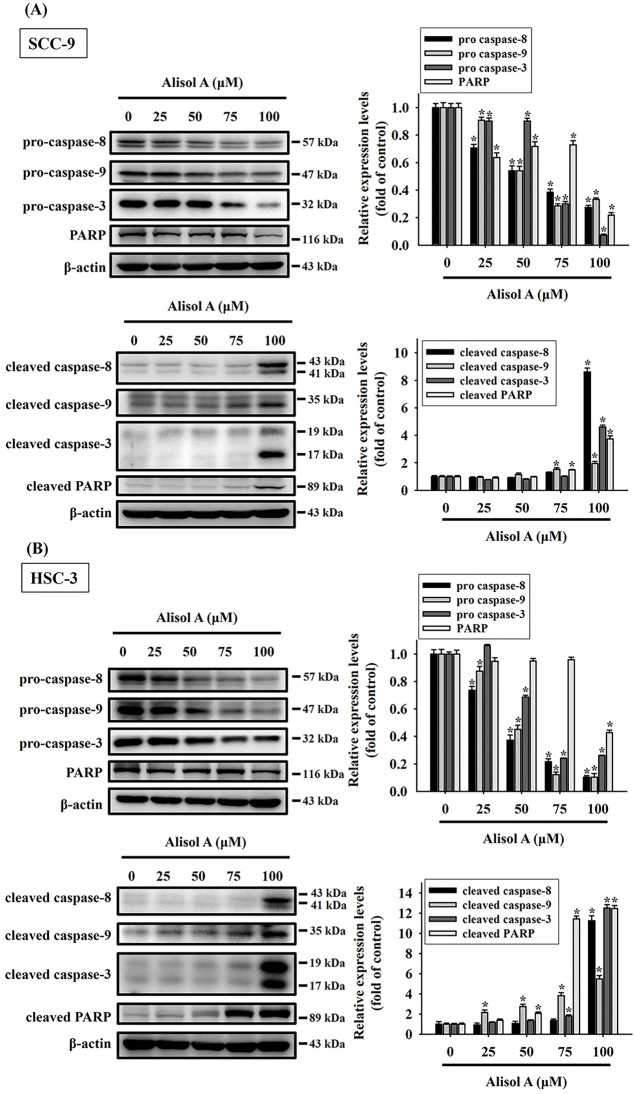
Alisol A contributed activation of caspase-3, -8, and -9 in human oral cancer cells. (**A**) SCC-9 and (**B**) HSC-3 cells were treated with serial concentrations of Alisol A (25, 50, 75, and 100 μM) for 24 h as indicated, and then the cells were subjected to immunoblotting to evaluate the levels of pro-caspases and cleaved caspases by immunoblotting. Data were performed using densitometric analysis and normalization with internal control β-actin, and presented quantitatively as mean ± SD from three independent experiments. *p < 0.05 vs. control

Given that the expression of apoptotic signaling proteins was regulated by Alisol A, the caspase-mediated cascade was subsequently explored. As shown in Fig. 5A, Alisol A significantly reduced the levels of pro-caspase-8, -9, -3, and PARP (*p* < 0.05 vs. control), while elevating the levels of cleaved caspase-8, caspase-9, caspase-3, and PARP in SCC-9 cells (*p* < 0.05 vs. control). These changes of caspases were dose-dependent, and the mean levels of cleaved caspase-8, -9, -3, and PARP were elevated up to 8.6-fold, 1.9-fold, 4.6-fold, and 3.7-fold of control, respectively. Similarly, levels of pro-caspases and cleaved caspases were reduced and elevated, respectively, in response to Alisol A treatments in HSC-3 cells (Fig. 5B, left panel), and the mean levels of cleaved caspase-8, -9, -3, and PARP were elevated up to 11.3-fold, 5.5-fold, 12.5-fold, and 12.5-fold of control, respectively (*p* < 0.05 vs. control, Fig. 5B, right panel). Collectively, the findings show that Alisol A results in activation of caspase-mediated cascade through mitochondrial and extrinsic pathways, consequently inducing cell apoptosis in oral cancer cells.

### 3.6 HO-1 Is a Critical Regulator Involved in Alisol A-Induced Apoptosis in Oral Cancer Cells

**Figure 6:**
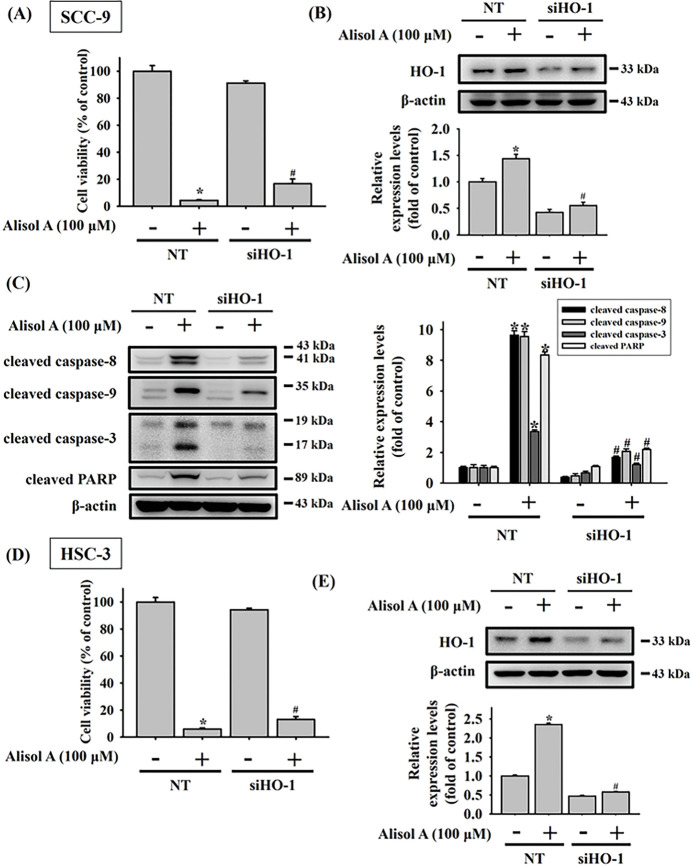

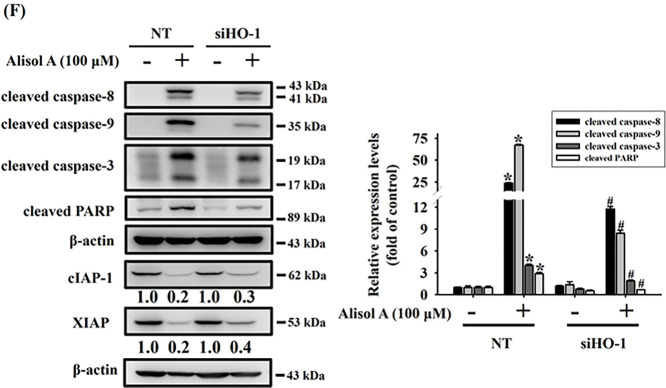
Upregulation of heme oxygenase-1 (HO-1) plays a pivotal role in Alisol A-triggered apoptosis through caspase activation in oral cancer cells. SCC-9 cells were transiently transfected with either non-targeting control siRNA (NT) or HO-1-specific siRNA. (**A,B**) The effects on cell viability and knockdown efficiency were analyzed by MTT assay and Western blotting, respectively. Silencing HO-1 counteracted the Alisol A-induced elevation of cleaved caspase-8, -9, -3, and PARP levels (**C**). Similarly, HSC-3 cells underwent transient transfection with control or HO-1 siRNA. (**D,E**) Subsequent cell viability and knockdown efficiency were examined via MTT and Western blot analysis. (**F**) HO-1 knockdown not only reversed the Alisol A-induced increases in cleaved caspase-8, -9, -3, and PARP, but also restored the Alisol A-suppressed expression of cIAP-1 and XIAP. *p < 0.05, compared to the vehicle group; ^#^*p* < 0.05 compared to the NT-transfected group

Recent studies have shown that HO-1 plays a paradoxical role across various types of malignancies [15,16]. In oral cancer, HO-1 has been implicated in apoptotic cell death induced by natural compounds [17]. To further investigate the role of HO-1 in Alisol A-induced apoptosis in oral cancer cells, we silenced HO-1 expression using specific HO-1 siRNA. Our results showed that silencing HO-1 significantly reversed the Alisol A-induced reduction in cell viability (Fig. 6A). Furthermore, we observed that the knockdown of HO-1 significantly reversed the Alisol A-induced upregulation of HO-1 protein, along with a concomitant downregulation of activated caspases-8, -9, and -3, as well as PARP in Alisol A-treated SCC-9 cells (Fig. 6B,C). Similarly, the cell viability and the levels of HO-1 were reversed in response to Alisol A treatment in HSC-3 cells (Fig. 6D,E). Moreover, HO-1 knockdown not only reversed the Alisol A-induced increases in cleaved caspase-8, -9, -3, and PARP, but also restored the Alisol A-suppressed expression of cIAP-1 and XIAP (Fig. 6F). Collectively, these findings suggest that the upregulation of HO-1 plays a critical role in Alisol A-induced apoptotic cell death in oral cancer cells.

### 3.7 Alisol A Provokes Activation of Caspases through JNK/p38 Signaling in Oral Cancer Cells

**Figure 7:**
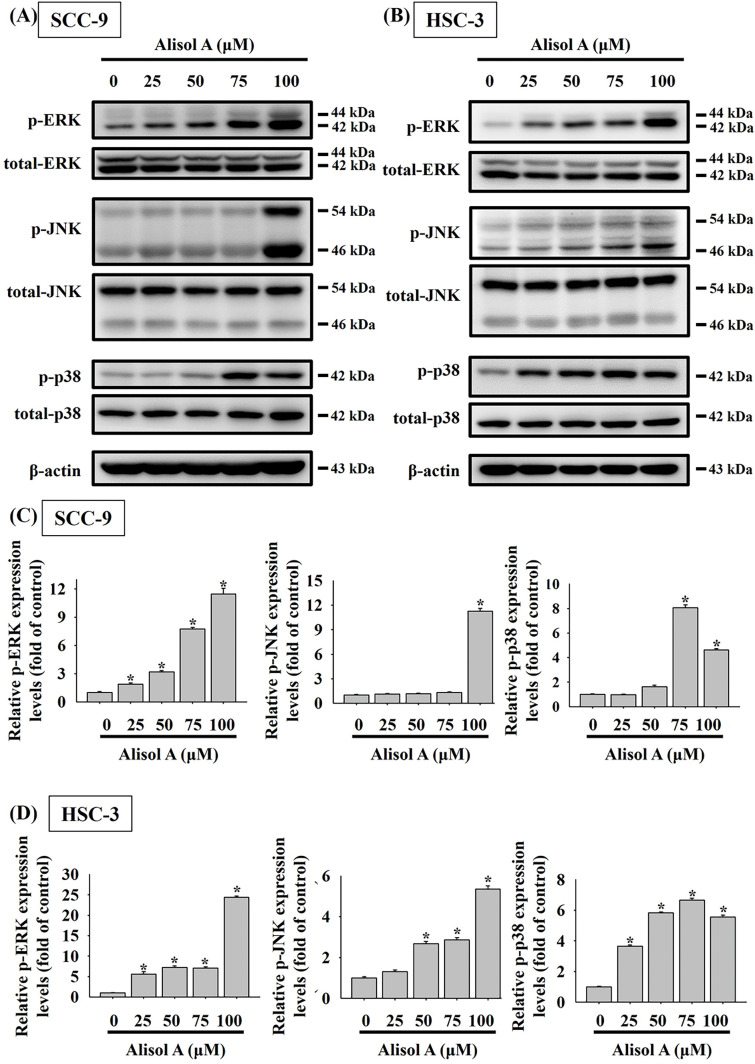
Alisol A provoked phosphorylation of ERK, JNK, and p38 in oral cancer cells. (**A**) SCC-9 cells and (**B**) HSC-3 cells were reacted with serial concentrations of Alisol A (25, 50, 75, and 100 μM) for 24 h, and then the cells were subjected to immunoblotting to assess the phosphorylated and total protein levels of ERK, JNK, and p38 using specific antibodies. (**C,D**) Chemiluminescence signals of Alisol A-induced phosphorylated MAPKs were quantified by densitometric analysis compared with controls. Data were expressed as mean ± SD from three independent experiments. *p < 0.05 vs. control

MAPK signaling has been informed to play a key role in cancer cell apoptosis in response to natural triterpenoids [18]. Therefore, the participation of MAPK signaling in Alisol A-induced apoptosis was explored. As shown in Fig. 7, Alisol A remarkably triggered phosphorylation of ERK1/2, JNK1/2, and p38 in both SCC-9 and HSC-3 cells. With the treatment of 100 μM Alisol A, the mean levels of phosphorylated ERK1/2, JNK1/2, and p38 were increased to 11.4-fold, 11.3-fold, and 4.6-fold of control, respectively, in SCC-9 cells (*p* < 0.05 vs. control, Fig. 7C). Consistently, the mean levels of phosphorylated ERK1/2, JNK1/2, and p38 were increased to 24.4-fold, 5.4-fold, and 5.6-fold of control, respectively, in HSC-3 cells with exposure to 100 μM Alisol A (*p* < 0.05 vs. control, Fig. 7D). Interestingly, among different concentrations of Alisol A treatment, 75 μM but not 100 μM induced the highest level of phosphorylated p38 in both oral cancer cells (Fig. 7A–D). These observations indicate that Alisol A differentially provokes activation of ERK1/2, JNK1/2, and p38 in oral cancer cells.

**Figure 8:**
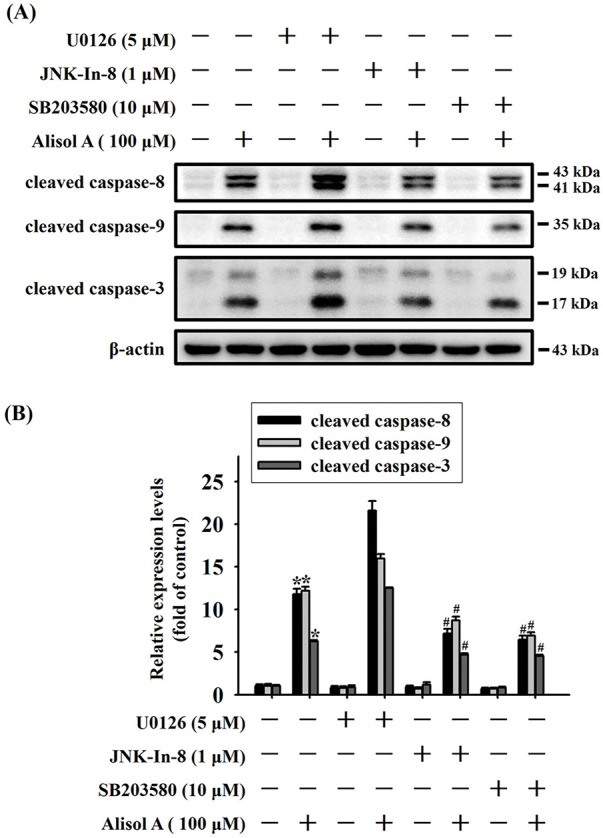
JNK and p38 activation are involved in Alisol A-induced caspase cascade in human oral cancer cells. (**A**) SCC-9 cells were preincubated with 5 μM U0126 (ERK inhibitor), 1 μM JNK-in-8 (JNK inhibitor), or 10 μM SB203580 (p38 inhibitor) for 2 h, reacted with 100 μM Alisol A for 24 h, and then subjected to immunoblotting to evaluate the level of cleaved caspase-3, -8, and -9. (**B**) Chemiluminescence signals of Alisol A-induced cleaved caspases were quantified by densitometric analysis compared with controls. β-actin was used as an internal control. Data were expressed as mean ± SD from three independent experiments. *p < 0.05 vs. control. ^#^*p* < 0.05 vs. Alisol A alone

Given that Alisol A provoked activation of MAPK pathway in oral cancer cells, the role of each MAPK in caspase activation in response to Alisol A was subsequently evaluated by using specific inhibitors. As shown in Fig. 8A,B, Alisol A increased levels of cleaved caspase-8, -9, and -3 in SCC-9 cells (*p* < 0.05 vs. control). Compared with Alisol A treatment alone, pretreatment of JNK inhibitor (JNK-IN-8) or p38 inhibitor (SB203580) significantly reduced the levels of cleaved caspase-8, -9, and -3 in SCC-9 cells stimulated with Alisol A (*p* < 0.05 vs. Alisol A alone). Notably, pretreatment of ERK inhibitor (U0126) followed by Alisol A treatment insignificantly affected the levels of cleaved caspase-8, -9, and -3 in comparison with Alisol A treatment alone (*p* > 0.05 vs. Alisol A alone, Fig. 8A,B). Collectively, these findings reveal that JNK/p38 signaling, but not ERK is involved in the caspase activation in oral cancer cells.

## 4 Discussion

OSCC is the most common malignant tumor in the oral cavity and has a high mortality and recurrence rate [3]. Recently, several natural compounds and their combination with chemodrugs have been reported to present potential anticancer effects on OSCC [19–21]. Among the four prostane-type triterpenes isolated from *Alismatis Rhizoma*, Alisol B is reported to exert significant cytotoxicity against epithelial cancer cells [22]. In line with these findings, our findings indicate that Alisol A shows anticancer activity against OSCC SCC-9 and HSC-3 cells by remarkably inhibiting their viability. In addition, our study also demonstrates that the inhibitory effect of Alisol A on OSCC cells is attributed to activation of the apoptotic cascade via JNK/p38-mediated signaling. This suggests that Alisol A possesses anticancer potential against OSCC cells and may facilitate current treatments for OSCC.

With the advances of early diagnosis and multiple therapeutic strategies, patient mortality has been reduced in certain types of cancer. However, relapse and drug resistance remain major challenges in cancer treatment. To improve the efficacy of current cancer treatments, combined treatment with radiotherapy, chemotherapy, and immunotherapy has emerged as a promising strategy. Previous studies have shown that Alisol A exhibits anticancer activity through modulating different signaling pathways, including inhibition of the Hippo signaling pathway, inactivation of PI3K/Akt signaling, and PI3K/Akt/mTOR axis [11–13,23]. In addition, Alisol A also has protective effects on neurovascular injury in cerebral ischemia [24] and high-fat diet-induced pathological brain aging [25]. Here, our results further reveal that Alisol A harbors anticancer potential against oral cancer cells via inducing JNK/p38-mediated apoptotic cascades. Taken together, these evidences suggest that Alisol A not only has therapeutic potential in developing combination therapies for oral cancer, but also may exert protective potential against chemotherapy-induced neuronal and vascular damage.

HO-1 plays a crucial role in catalyzing the breakdown of heme into CO, free iron, and biliverdin. HO-1 is also an important antioxidant enzyme that protects against oxidative stress, and therefore, intracellular oxidative stress induces expression of HO-1 [26]. Our observations show that Alisol A upregulates HO-1 expression in oral cancer cells, implicating that Alisol A may induce intracellular oxidative stress and subsequently promote HO-1 expression. However, HO-1 not only exerts cytoprotective effects on normal cells, but also on tumor cells. High expression of HO-1 has been reported in different cancers and is associated with poor prognosis, including esophageal squamous cell carcinoma [27], neuroblastoma [28], and breast cancer [29]. Thus, previous studies show that targeting HO-1 may be a promising strategy to promote cancer treatments and reduce chemoresistance [30–32]. Notably, the association between HO-1 expression and oral cancer is rarely explored. Moreover, Tsuji et al. report that high HO-1 expression in OSCCs can be useful in identifying patients at low risk of lymph node metastasis [33]. Collectively, the role of HO-1 in oral cancer, at least OSCC, remains sketchy and requires further investigation.

High expression of IAPs, an E3 ubiquitin ligase family, in cancers has been reported to associate with cancer cell survival [34]. Previous reports indicate that IAPs trigger ubiquitin-mediated degradation of caspases-3, -7, and -9, and consequently inhibit caspase-mediated cell apoptosis [34,35]. In contrast, apoptosis-inducing signals lead to the release of IAP-binding proteins such as SMAC/DIABLO, which trigger apoptosis by binding to IAP and causing IPA degradation [36]. IAPs have also been demonstrated to suppress Bax expression and apoptosis induced by chemotherapy or ultraviolet radiation [37,38]. Additionally, SM-164, which inhibits IAP proteins, has emerged as a potential treatment for early-stage bone and lung metastases in triple-negative breast cancer.r [39]. Our observations revealed that Alisol A remarkably diminished the levels of cIAP1 and XIAP, suggesting that inhibition of cIAP1 and XIAP may be involved in the Alisol A-provoked apoptosis of oral cancer cells. Nevertheless, the mechanistic act to upregulate cIAP1 and XIAP by Alisol A still needs further investigation.

MAPKs belong to a family of protein kinases and play a critical role in transducing intracellular signals that govern important cellular responses in response to extracellular stimuli [40]. Therefore, MAPK signaling is widely involved in the mechanistic actions induced by chemotherapeutic agents [41]. JNK signaling has been reported to be involved in cancer cell apoptosis in response to natural compounds [42]. Similarly, p38 activation is also broadly associated with phytochemical-induced cancer cell death [43]. In addition, certain phytochemicals combined with chemodrugs synergistically promote anticancer effects mainly through activating the JNK/p38 signaling pathway [44,45]. In line with our findings, Alisol A induces caspase activation in oral cancer cells through the JNK/p38-mediated cascade, suggesting that JNK/p38 signaling plays an important role in Alisol A-induced apoptosis.

This study has several limitations. First, cytotoxicity data in normal epithelial cells were not included, which limits our ability to fully assess the safety profile of Alisol A in non-cancerous contexts. Future studies will address this by evaluating the effects of Alisol A in normal epithelial cell lines. Second, *in vivo* validation of the combination therapy involving Alisol A and an ERK inhibitor was not performed. Further investigation using appropriate animal models will be necessary to determine the therapeutic efficacy and safety of this combination *in vivo* and to strengthen its translational relevance. Furthermore, combination therapy approaches, such as co-treatment with current chemotherapeutic agents or targeted inhibitors, may reveal potential synergistic effects and help overcome drug resistance in oral cancer.

**Figure 9:**
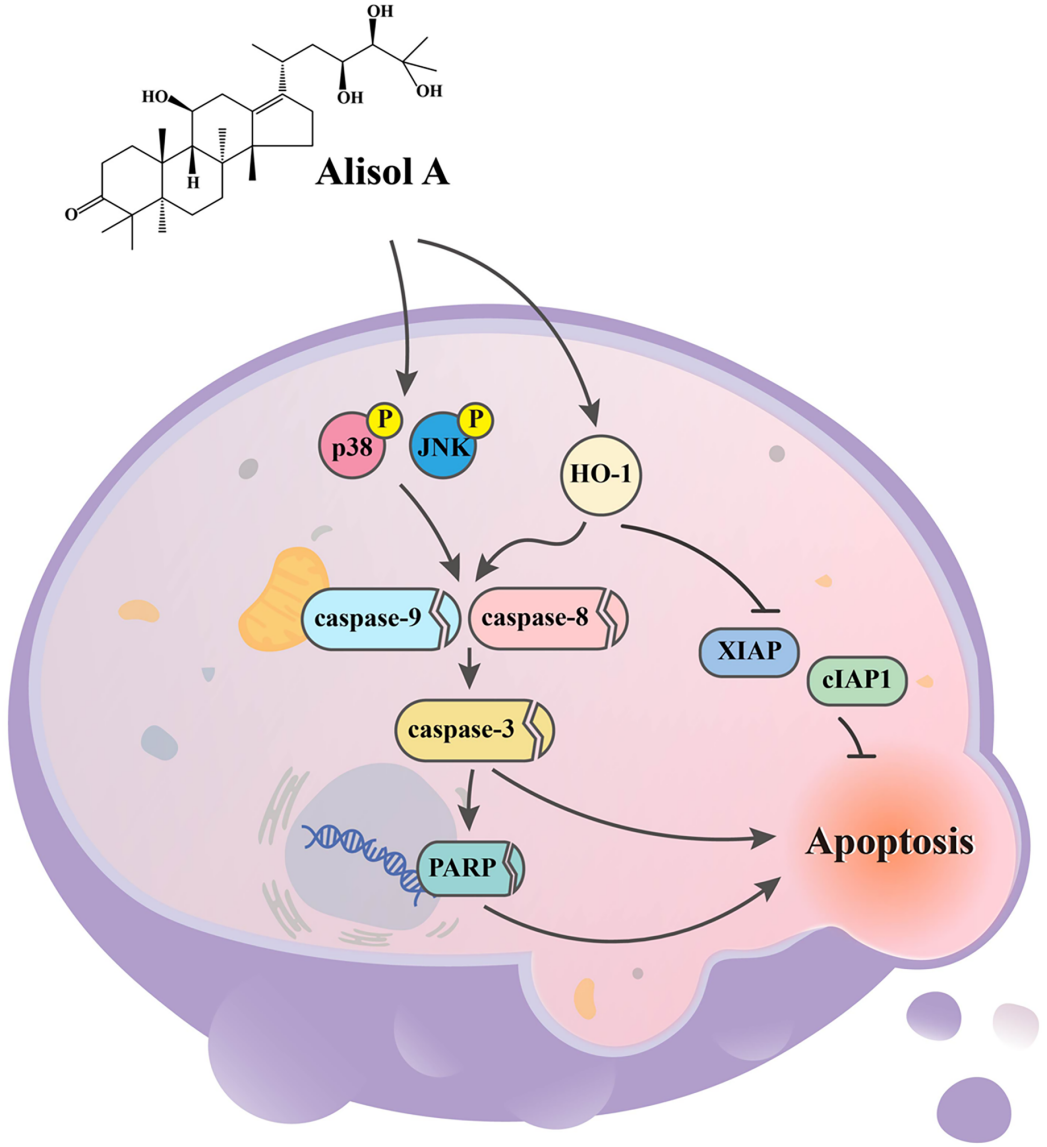
Working model showing the proposed signal transduction pathways underlying the ability of Alisol A to inhibit the growth of oral cancer cells. Our results show that Alisol A remarkably diminishes the viability of SCC-9 and HSC-3 cells, which may be attributed to JNK/p38-mediated apoptotic cascade activation and the consequent cell apoptosis

In the present study, we evaluate the anticancer effect of Alisol A on human oral cancer cells. Our results show that Alisol A remarkably diminishes the viability of SCC-9 and HSC-3 cells, which may be attributed to JNK/p38-mediated apoptotic cascade activation and the consequent cell apoptosis (Fig. 9). These findings indicate that Alisol A harbors potential anticancer capability against oral cancer cells. Accordingly, it suggests that Alisol A might help promote the efficacy of oral cancer treatment.

## Data Availability

The data used to support the findings of the present study are available from the corresponding authors upon request.
